# A semi-automated workflow for biodiversity data retrieval, cleaning, and quality control

**DOI:** 10.3897/BDJ.2.e4221

**Published:** 2014-12-11

**Authors:** Cherian Mathew, Anton Güntsch, Matthias Obst, Saverio Vicario, Robert Haines, Alan R. Williams, Yde de Jong, Carole Goble

**Affiliations:** † Freie Universität Berlin, Botanic Garden and Botanical Museum Berlin-Dahlem, Berlin, Germany; ‡ Department of Biological and Environmental Sciences, University of Gothenburg, Gothenburg, Sweden; § Institute of Biomedical Technologies, National Research Council, Bari, Italy; | School of Computer Science, University of Manchester, Manchester, United Kingdom; ¶ University of Eastern Finland, Joensuu, Finland; # University of Amsterdam - Facilty of Science, Amsterdam, Netherlands

**Keywords:** biodiversity informatics, web services, workflows, service oriented architecture, data cleaning, e-Science

## Abstract

The compilation and cleaning of data needed for analyses and prediction of species distributions is a time consuming process requiring a solid understanding of data formats and service APIs provided by biodiversity informatics infrastructures. We designed and implemented a Taverna-based Data Refinement Workflow which integrates taxonomic data retrieval, data cleaning, and data selection into a consistent, standards-based, and effective system hiding the complexity of underlying service infrastructures. The workflow can be freely used both locally and through a web-portal which does not require additional software installations by users.

## Introduction

Over the last decade, the international biodiversity informatics community has built service-oriented infrastructures that provide constantly updated datasets and computational services supporting the mobilisation (data gathering), compilation (data structuring), contextualization (data standardisation based, for example, on TDWG Biodiversity Information Standards, www.tdwg.org), integration and validation (data cleaning) of species-related occurrence information (Berendsohn et al. 2011). However, these services are not generally used by scientists who in most cases still prefer to prepare their datasets manually, using spreadsheets or their own (local) databases. Although infrastructures such as GBIF (www.gbif.org) and BioCASE (www.biocase.org) offer consistent interfaces to many distributed occurrence and taxon-level datasets, a substantial part of the data resources required for scientific experiments are not organized in, or connected to, such global systems and instead provide separate, inaccessible, and incompatible interfaces and data formats. In addition, software tools and services for data enrichment and quality control are likewise diverse and there is no comprehensive registry available for scientists to discover and deploy services that access and integrate relevant biodiversity data over large taxonomic, spatial, and temporal scales.

A range of data services and tools have been developed to address data aggregation, cleaning and curation aspects such as Kurator (Dou et al. 2012) and data quality control mechanisms in EurOBIS (www.eurobis.org). Here we present an approach to improve the effectiveness of species occurrence data compilation using scientific workflows implemented with the Taverna Workflow Management System (Wolstencroft et al. 2013). We designed and implemented a Taxonomic Data Refinement Workflow that integrates services needed for occurrence retrieval, enrichment, cleaning, refinement, and filtering of taxonomic data. The workflow is designed to support scientists in performing interdisciplinary and complex analytical tasks, arising from the combination of disparate services, both local and remote, each with their own input parameters and output formats. In this design each workflow element represents a specific service, with its own input and output ports, which in turn plugs in to other elements. This modular construction allows software developers to build complex analytical processes by successively composing and testing individual sections, which are subsequently added to the workflow. The obvious advantage is that users can concentrate on their own domain (e.g. taxonomy, ecology) without being exposed to the specific technical requirements of all the domains that are a part of the workflow (e.g. service interface specifications, GIS input requirements).

## Project description

### Study area description

**Figure 1.**
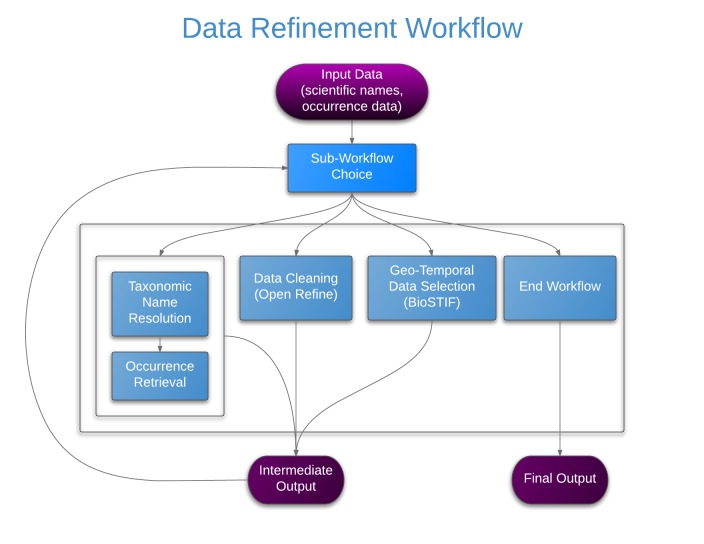
Taxonomic Data Refinement Workflow. Schematic diagram showing the integrated functions. Intermediate output from each section of the workflow can be stored and re-used as input for subsequent iterations.

The Taxonomic Data Refinement Workflow integrates a range of services to perform data processing tasks with the possibility of adding new services if required. The workflow accepts species occurrence data, taxon-level data, or a list of scientific names as input, and offers three distinct sub-workflows for particular tasks depending on the input type (Fig. 1). These are

**Figure 2.**
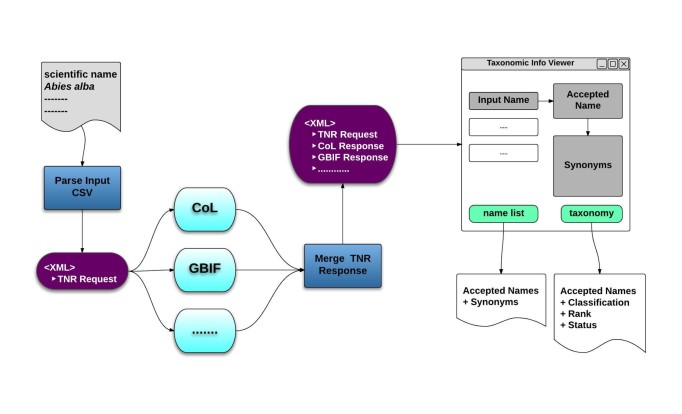
Taxonomic Name Resolution. Overview of the Name Resolution function of the Taxonomic Data Refinement Workflow, depicting the aggregation of scientific name responses from the various checklist into a single XML message. This message is then used to display the results within a web interface.

**Figure 3.**
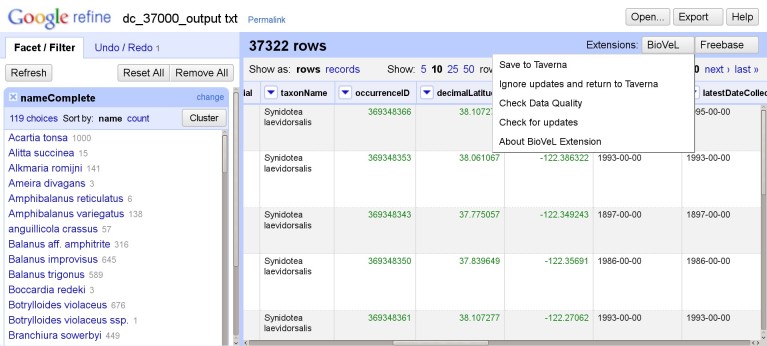
OpenRefine interface with the BioVeL extension. The extension adds biodiversity data specific functionality to OpenRefine for the purposes of data cleaning, integration, and refinement. The GoogleRefine branding in the screenshot is due to the fact this workflow uses the last stable released version (2.5) of OpenRefine when the software was still being developed by Google.

**Figure 4.**
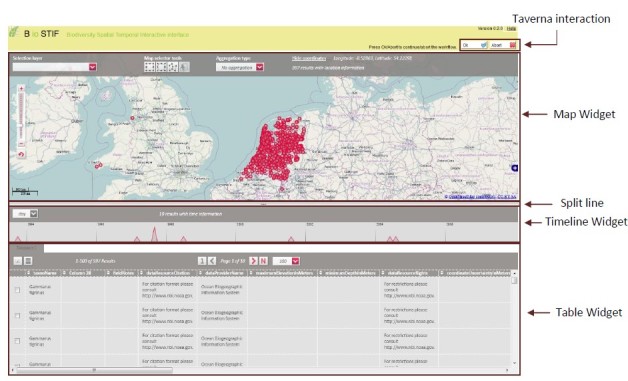
BioSTIF web interface. The interface allows users to filter species occurrence points based on selected geographical regions and time periods.

1. Taxonomic Name Resolution and Occurrence Retrieval: Scientific names included in the input dataset are submitted to user-selected checklists (Fig. 2). Users browse through the respective lists of accepted names and synonymies and choose names that will be added to the original input list. Users then retrieve species occurrence records from selected occurrence data services and the retrieved datasets are then added to the original user data. Depending on how correct, complete and up-to-date the checklist services are, the quality of responses used as a basis for name resolution may vary considerably. The decision on the choice of responses to be included in the resolution process for individual names has to be taken by the user;
2. Data Cleaning: Cleaning of data records as well as semantic enrichment are conducted using a biodiversity-specific extension of OpenRefine (http://openrefine.org/) (Fig. 3). Typical activities include the mapping of data fields to controlled vocabularies (standardised taxonomies), resolving nomenclatural variations, and supporting the identification and exclusion of erroneous or irrelevant records. Targeted checklists can be selected at different stages of the name resolution process (Fig. 6);
3. Geo-Temporal Data Selection: Users filter records in time and space using the BioSTIF system (https://wiki.biovel.eu/display/doc/BioSTIF+User+Manual) which offers an interactive GIS-like interface for selecting records by drawing polygons on a map and by defining time slices (Fig. 4).

Inputs and outputs of each section are compatible and users may execute sections in any order and as many times as needed, with the option to end the workflow at any point.

Traditionally, workflows are used for automating complex or large-scale data processing tasks, often requiring systematic and multiple analyses over sets of data or parameters. However, the power of the Taxonomic Data Refinement Workflow lies in its flexible access to highly specialized and distributed services without exposing the computational protocols needed to interact with them; and the structuring of the studies into a systematic protocol whose results can be compared, its process documented and the source of the results logged. The seamless integration of these service functions enables scientists to inspect large biodiversity data sets simultaneously from different angles (e.g. taxonomic, geographic, ecological), and this integrated view allows for appropriate data selection that leads to the generation of comparable data sets.

### Design description

The workflow has been constructed using Taverna Workbench (http://www.taverna.org.uk/download/workbench/) by progressively building each section using individual modules called ‘services’. A number of frequently used services are already available within the workbench environment and developers are encouraged to build their own specific services using Beanshell scripting (http://jcp.org/en/jsr/detail?id=274). This allows for the creation of complex analysis pipelines connecting various local and remote services and nested workflows. Once constructed, the workflows are reusable executable biodiversity informatics protocols that can be shared, reused and repurposed. One of the main features of the workflow is the web browser-based interaction inspired by the development of the Interaction plugin (http://www.taverna.org.uk/documentation/taverna-2-x/interaction) for the Taverna environment. The browser-based approach opens up the possibility of running the workflow on Taverna Player (http://www.taverna.org.uk/documentation/taverna-2-x/taverna-player), a remote execution environment with web-based interactions, which eliminates the requirement to download and install any local software components. This implies that the workflow can be executed both locally and within a remote server environment.

**Workflow complexity**

**Figure 5.**
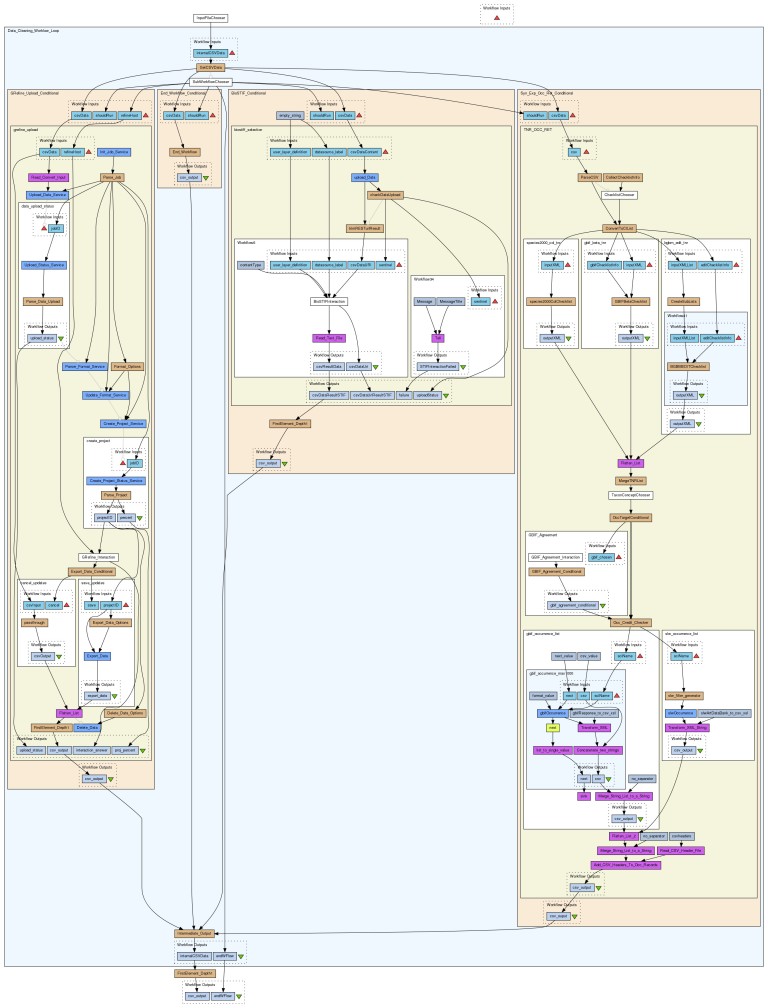
Data Refinement Workflow. Birds eye overview of service interactions of the workflow as shown in Taverna Workbench.

**Figure 6.**
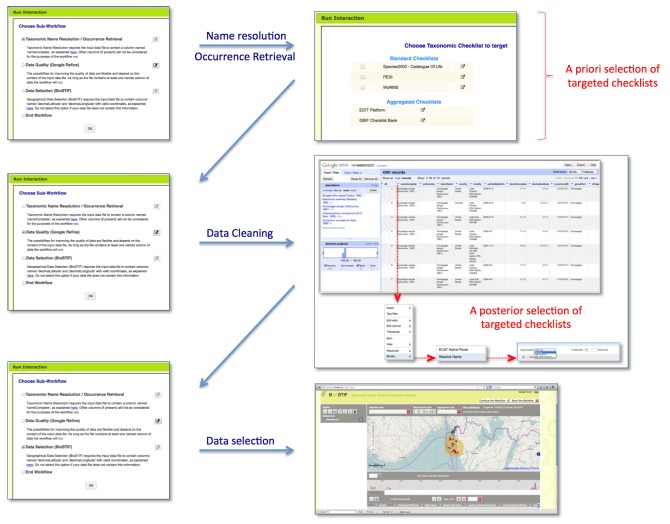
Selection of targeted checklists (as controlled taxonomic vocabularies) in the name resolution process.

Converting the conceptual idea of the workflow into an automated form has revealed the complexity inherent in combining various kinds of services, both local and remote, each with their own input parameters and output formats. (Fig. 5). Each element represents a specific service, with its own input and output ports, which in turn plug in to other elements. The ability to build workflows by connecting together compatible elements allows software developers to construct the workflow by building and testing individual sections, before integrating them into the final workflow. This modular nature of the development process is also beneficial when considering that such workflows are usually built in close collaboration with scientists. This methodology encourages an agile working environment where scientists can easily test and provide feedback on individual components, which are then used by the developers to further enhance the workflow.

**Workflow design**

**Workflow input format:** The workflow accepts input data from a user supplied CSV (http://tools.ietf.org/html/rfc4180) file, with controlled header terms referring to concepts defined by TDWG LSID Vocabularies (http://wiki.tdwg.org/twiki/bin/view/TAG/LsidVocs). These vocabularies, in particular the TaxonName (http://wiki.tdwg.org/twiki/bin/view/TAG/TaxonNameLsidVoc) and TaxonOccurrence (http://wiki.tdwg.org/twiki/bin/view/TAG/TaxonOccurrenceLsidVoc) subset have been chosen because they represent a set of well documented vocabularies (https://wiki.biovel.eu/display/doc/Preparing+your+input+data+for+Data+Refinement) defined for common concepts in the biodiversity informatics domain. Records in the CSV input file must at least consist of either,

1. a list of taxonomic names made up of TaxonName information
2. a set of occurrence observation records comprising both TaxonName and TaxonOccurrence information.

The decision to support only the CSV format with a restricted set of header terms has kept the development effort focused on quickly adding various functionality to the workflow rather than making the workflow compatible with different types of existing input formats and vocabularies. This decision implies that the input data preparation for the workflow needs to be done by the user. However, by keeping the format simple and limiting the number of terms, manual intervention is minimized. Future versions of the workflow will support additional data input formats, which can be transformed into a normalized format using transformation software such as Pentaho Kettle (http://sourceforge.net/projects/pentaho/).

**Taxonomic name resolution and species occurrence retrieval:** Resolving names to taxonomic concepts is one of the most crucial functionalities of the workflow (Güntsch et al. 2009). The objective of this section is to expand a list of scientific names into corresponding taxonomic concepts and then extract relevant information from these concepts. The retrieved information includes classification, synonymy, original source details and taxonomic scrutiny. The process expects input in the format described above, which is initially parsed by a CSV parser to extract scientific names. The names are then transformed into a structured and generic XML-based representation encapsulating the different conventions used by the various taxonomic checklists and integrating them into a single unit. Once the input data are parsed, a request element corresponding to each scientific name is added to the XML representation. This request is then transformed into specific REST API calls to a number of online taxonomic checklists that provide web services to resolve scientific names. These resolution services map the input names to related taxonomic information including synonymies, classification hierarchies, taxonomic status, rank, etc. and were chosen based on scientific requirements of the pilot users. Currently, the list of targeted checklists include the,

- Catalogue of Life (CoL; Roskov et al. 2014; www.catalogueoflife.org/),
- Global Biodiversity Information Facility Checklist Bank (GBIF; http://www.gbif.org/species),
- EDIT Platform for Cybertaxonomy (EDIT; Berendsohn 2010; http://cybertaxonomy.eu/cdmlib/rest-api-name-catalogue.html),
- World Register of Marine Species (WoRMS, http://www.marinespecies.org/), and
- Pan-European Species directories Infrastructure (PESI, http://www.eu-nomen.eu/portal/).

with more planned for the future. The response from each of these web service calls is converted to XML form and appended to the corresponding request. The final representation can be then visualized in a viewer, which displays the relationship between names and their corresponding taxonomic concepts, with the possibility of extracting specific information. Currently the options include the generation of a de-duplicated list of accepted names and their synonyms as well as a list of accepted names along with corresponding taxonomic information.

The option to retrieve occurrence records of species obtained as a result of name resolution is also provided within this section of the workflow. Currently the only target is the GBIF Occurrence API (http://www.gbif.org/occurrence), which provides a single access point to more than 500 million distributed occurrence records. Additional targets, equipped with a standardized service interface, can be included as per requirements.

This workflow section provides an interface for functionality related to taxonomic data aggregation and this aspect can be greatly improved in the future. For instance, the name resolution section works only with exact name matches and does not provide any kind of reconciliation feature which most experts may consider as a crucial requirement. Another feature that would make the workflow more efficient is to expose specific data provider options to the user, e.g. allowing the user to select geographical bounds to the occurrence data requested from providers like GBIF which offer this kind of capability.

**Taxonomic Data Cleaning:** The quality of taxonomic data and species occurrence records plays an important role in biodiversity analysis. Given that loss of data quality could occur in any of the multiple stages of data collection, it is of utmost importance that the data retrieved from the various sources be ‘fit for use’ in relation to the study undertaken (Chapman 2005). There already exist a number of specialized tools, libraries and applications, which perform data cleaning on various types of data. The main objective of this section of the workflow is to provide a semi-automated user interface environment with data cleaning features specific to taxonomic data sets. These features can be divided into three main categories,

1. Data Quality Checks: This includes a global quality check on specific elements of the dataset (e.g. validity of latitude / longitude values, date validation, etc).
2. Data Transformation: This set of features allow for the conversion of data into a form which is fit for purpose (e.g clustering scientific names using name parsers, conversion of data units, etc.).
3. Data Extension: This category of features makes it possible to enrich existing data by using local and remote services (e.g. resolving scientific names to their accepted names, reverse geo-referencing, etc.).

Following initial investigations on the feasibility of building such an environment based on existing solutions, it was decided to use OpenRefine. In addition to the intuitive interface with the various faceted views to edit data, the ability to extend functionality was a crucial factor in the decision. This has led to the implementation of the BioVeL Extension which integrates custom-made functionality, third-party libraries and remote services to provide features which cover the three categories mentioned above. OpenRefine along with the BioVeL Extension also shows considerable potential in other use cases such as the cleaning of taxonomic data prior to upload into managed databases as well as playing a role in the annotation of already existing data. All data processing activities can be recorded in JSON format for re-use on different data sets or as a data processing log (www.ietf.org/rfc/rfc4627.txt)

**Geo-Spatial / Temporal Data Selection:** This section of the workflow deals with the selection of data based on geographical regions as well as the filtering of records with respect to time-based information. The selection is performed using the geo-server based, web-enabled, GIS application BioSTIF (www.biodiversitycatalogue.org/services/7). The tool allows the user to filter in/out occurrence data points by constructing polygonal regions using the given toolbox. A temporal range can also be selected by using the timeline tool to choose a specific period of interest. Along with these functionalities the tool also provides a tabular view of the data.

**The workflow in use**

The Data Refinement Workflow has been used in a number of scientific studies including

1. Leidenberger S, De Giovanni R, Kulawik R, Williams AR, Bourlat SJ (2014) Mapping present and future potential distribution patterns for a meso-grazer guild in the Baltic Sea (http://onlinelibrary.wiley.com/enhanced/doi/10.1111/jbi.12395/)
2. Laugen et al (In Review) The Pacific Oyster (*Crassostrea gigas*) invasion in Scandinavian coastal waters in a changing climate: impact on local ecosystem services. In Biological Invasions in Aquatic and Terrestrial Systems: Biogeography, Ecological Impacts, Predictions, and Management. De Gruyter, Warsaw.
3. Leidenberger S, Obst M, Kulawik R, Stelzer K, Heyer K, Hardisty A, Bourlat SJ (In Review) Evaluating the potential of ecological niche modelling as a component in invasive species risk assessments. Ecological Applications.

**User access and documentation**

The full workflow documentation (Data Refinement Workflow v.13) as well as an extensive tutorial is available at https://wiki.biovel.eu/display/doc/Data+Refinement+Workflow. The workflow can be downloaded from myExperiment under http://www.myexperiment.org/workflows/2874/versions/16.html, and executed through the BioVeL portal under http://portal.biovel.eu. Web-service documentations are accessible from the BiodiversityCatalogue (https://www.biodiversitycatalogue.org/).

**Outlook**

The Data Refinement Workflow presented in this paper is a generic approach to provide tools to end users working with biodiversity data. The solution presented here has been developed as an automated workflow and tackles problems related to the aggregation, cleaning and geo-temporal selection of data. Even though such tools have been traditionally developed by data service providers, in most cases these can be applied only to data specific to the providers. The DRW aims to enable users to aggregate and normalise data from various sources and then work on making the data fit-for-purpose for a target research use case.

The development of such kinds of workflows to be used in conducting in-silico experiments has exposed a number of technical issues. Firstly, it is becoming increasingly clear that a certain level of software expertise is required to develop automated workflows and efforts should be made to ease the technical burden on users (primarily scientists) who may not be so proficient in software development. For example, in its current form the Data Refinement Workflow restricts input data to the CSV format using controlled vocabularies, but should definitely allow for other formats (e.g. TSV, XML, etc) if the workflow approach is to be widely adopted.

The workflow has already been used in scientific research use cases which have greatly benefited from the use of the functionality and the efficiency provided by the workflow approach. Future work should include studies to quantify the level of benefit for the users and consider benchmarking the workflow approach as a whole and individual components in particular using well described metrics, which allow for a more objective view on how the DRW compares to other existing tools.

### Funding

The design and implementation of the Data Refinement Workflow was funded by the *EU’s Seventh Framework Program project* BioVeL (www.biovel.eu) *with the grant no. 283359.*

## Web location (URIs)

Homepage: https://portal.biovel.eu/

Wiki: https://wiki.biovel.eu/display/doc/Data+Refinement+Workflow

Download page: http://www.myexperiment.org/workflows/2874/versions/16.html

## Usage rights

### Use license

Creative Commons CCZero
